# Keratoacanthoma of the Nasal Septum Secondary to Ranibizumab Use

**DOI:** 10.1155/2017/8257590

**Published:** 2017-05-11

**Authors:** Jason E. Cohn, Hilary M. Caruso Sales, Giang Huong Nguyen, Harvey Spector, Kenneth Briskin

**Affiliations:** ^1^Department of Otolaryngology-Head and Neck Surgery, Philadelphia College of Osteopathic Medicine, 4190 City Line Avenue, Philadelphia, PA, USA; ^2^Department of Dermatology, University of Colorado Anschutz Medical College, 1665 Aurora Court, Aurora, CO, USA; ^3^Department of Pathology, Crozer-Chester Medical Center, One Medical Center Boulevard, Upland, PA, USA; ^4^Department of Otolaryngology-Head and Neck Surgery, Crozer-Chester Medical Center, One Medical Center Boulevard, Upland, PA, USA

## Abstract

Keratoacanthoma (KA) is a benign epithelial tumor that typically presents as a firm, cone-shaped, flesh-colored nodule with a central horn-filled crater. KA is considered to be a low-grade variant of squamous cell carcinoma (SCC). We report a rare case of a 72-year-old male who presented with a KA involving the nasal septum, possibly related to ranibizumab use. A flesh-colored lesion on the right anterior nasal septum lesion was visualized on examination. Histologic examination revealed a well-circumscribed, dome-shaped central crater filled with keratin, well-differentiated squamous epithelium with ground-glass cytoplasm with pushing margins, and intraepithelial microabscesses establishing the diagnosis of KA. KA of the nasal septum has only been reported once in the literature. This case is unusual because it normally presents on sun-exposed areas. Additionally, this patient was taking ranibizumab, a vascular endothelial growth factor (VEGF) inhibitor for macular degeneration. Despite ranibizumab not being directly linked to precancerous and cancerous skin lesions, agents in this medication class have been. Although it is difficult to prove associations in this isolated case, the role of ranibizumab causing cutaneous lesions should be further investigated.

## 1. Introduction

Keratoacanthoma (KA) was first described by Hutchinson as a “crateriform ulcer of the face” [1]. However, Freudenthal is credited for the term keratoacanthoma based upon acanthosis seen on histology [2]. KA is a benign epithelial tumor originating from pilosebaceous glands (hair follicles) that typically presents as a firm, cone-shaped, flesh-colored nodule with a central horn-filled crater in sun-exposed regions of middle-aged to elderly individuals [3, 4]. KA is considered to be a low-grade variant of squamous cell carcinoma (SCC) due to its rapid growth and histologic appearance and, because of this, wide surgical excision has often been the treatment of choice [2, 5]. Classically it will grow to 1-2 centimeters (cm) and spontaneously involute; however, there are unusual giant variants that can grow to larger than 2 cm [5–8]. The clinical course of KA has been described in 3 stages: proliferative, mature, and involutional. The proliferative stage starts with a firm, smooth, enlarging papule that rapidly grows over a 2–4-week period. It then progresses to a mature form described as a dome-shaped, skin-colored nodule with a central, often umbilicated, keratinous core. After several months, involution tends to occur characterized by tumor resorption and explosion of the central keratotic plug resulting in a slightly depressed, often hypopigmented scar [2]. The true differentiating factor between KA and SCC is this spontaneous involution of the KA; however, watching the lesion is deemed unadvisable and excision is frequently recommended before involution occurs [2].

## 2. Case Report

A 72-year-old Caucasian male presented to an outpatient practice with an approximate 1-year history of a right nasal lesion. He stated that it was cosmetically bothersome to him and requested evaluation. Associated symptoms included mild nasal obstruction, mild pain at the lesion site, and nasal congestion. Patient denied ulceration, bleeding, and drainage. Past medical history included seasonal (pollen) allergic rhinitis, macular degeneration, glaucoma, hypertension, and hyperlipidemia. Past surgical history included carpal tunnel release and meniscal tear repair. His family history was noncontributory and negative for skin disease. Social history revealed no use of tobacco, alcohol and illicit drugs. Current medications included pitavastatin, losartan, amlodipine, omeprazole, naproxen, and ranibizumab.

Vital signs obtained in the office were stable and within normal limits. The physical examination was only significant for a right anterior nasal septum lesion. It was well-circumscribed, flesh-colored, dry, and mildly tender to palpation. There was no ulceration or drainage appreciated. The lesion appeared to be localized with no extension. Cranial nerve examination was unremarkable. No other lesions or lymphadenopathy were noted on examination.

The decision was made to bring the patient to the operating room for wide local excision of this lesion. Examination under local anesthesia revealed a well-circumscribed, flesh-colored, crateriform lesion along the anterosuperior portion of the right nasal septum. Full excision of the lesion was performed and the surgical specimen was sent to the pathology laboratory for review. Gross examination revealed a 0.6 × 0.3 cm lesion which contained a centrally located ovoid papule measuring 0.3 × 0.3 × 0.2 cm.

**Figure 1:**
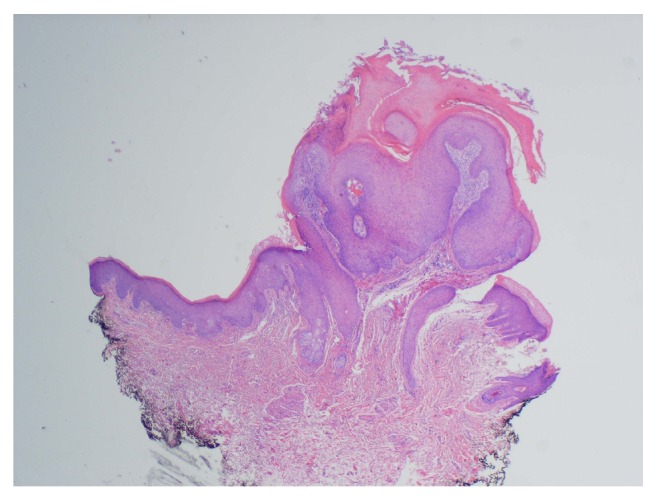
A well-circumscribed, dome, or cup-shaped central crater filled with keratin.

**Figure 2:**
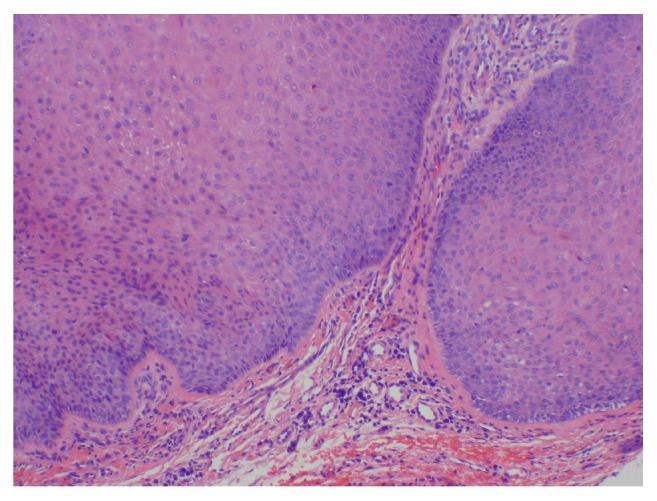
Pushing, noninfiltrating margin of squamous epithelium.

**Figure 3:**
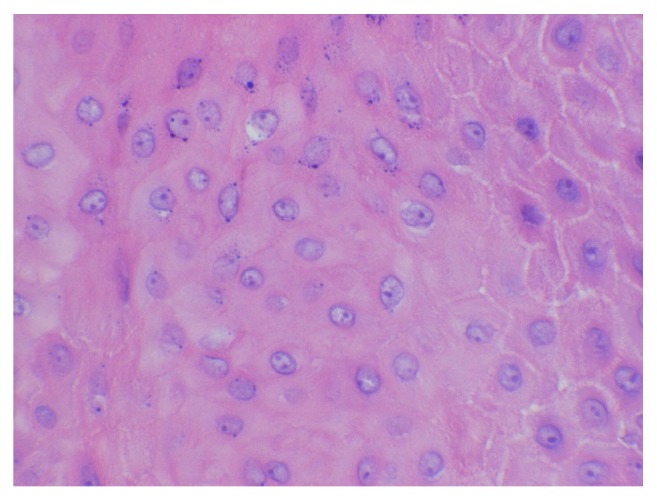
Well-differentiated squamous epithelium with ground-glass cytoplasm without atypia, dysplasia, or viral cytopathic effect.

**Figure 4:**
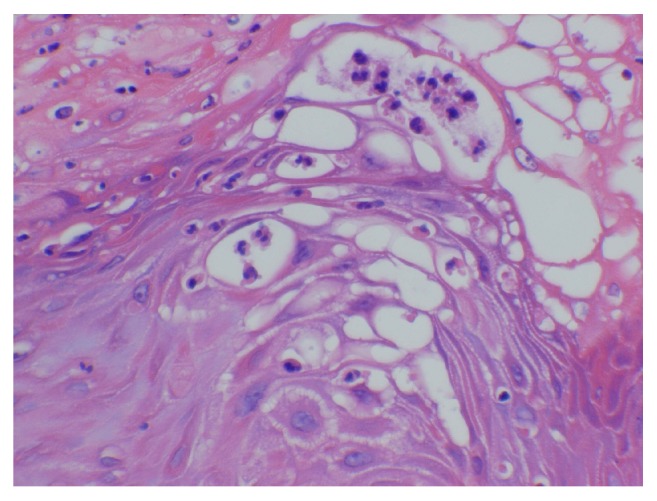
Intraepithelial microabscesses.

Histologic examination of the lesion revealed a well-circumscribed, dome-shaped central crater filled with keratin (Figure 1). A pushing margin of squamous epithelium was seen; however, it was noninfiltrating distinguishing it from squamous cell carcinoma (Figure 2). Well-differentiated squamous epithelium was observed with ground-glass cytoplasm (Figure 3), with no atypia, dysplasia, or viral cytopathic effect. Intraepithelial microabscesses were also present (Figure 4). These were several, classic findings consistent with the diagnosis of KA. Upon follow-up, the patient was pleased with the result and reported resolution of his previous symptoms with the exception of nasal congestion which is chronic for him. The patient was provided with the diagnosis and was given appropriate follow-up.

## 3. Discussion

KA is a rapidly growing cutaneous neoplasm most commonly seen on the face, forearms, legs, and hands. It usually affects light-skinned, middle-aged to elderly individuals, with a peak incidence between the ages of 50 to 69 years. Its time course often spans 4 to 6 months from origin to spontaneous involution but some may persist for over one year [2]. KA of the nose has been described to involve the external nasal structures [7–11] and less commonly the nasal vestibule [12, 13]. However, there has only been one report involving the nasal septum, which was in France nearly 40 years ago [14]. As mentioned previously, involvement of the nasal septum is unusual because KA normally affects sun-exposed areas.

Broadly speaking, the differential for a skin lesion of the nose can be divided into nonmalignant tumors, inflammatory conditions, vascular lesions, premalignant lesions, and malignant tumors. Nonmalignant tumors would include freckles, comedones, adenoma sebaceum, hidrocystoma, fibrous papule, sebaceous hyperplasia, and rhinophyma. Inflammatory conditions affecting the nose include pemphigus, sarcoidosis, systemic lupus erythematosus, facial eosinophilic granuloma, rosacea, herpes zoster, leishmaniasis, and leprosy. Vascular lesions observed in the nose are telangiectasia, hemangioma, and spider nevus. Premalignant lesions involving the nose include actinic keratosis and keratoacanthoma. Finally, malignant tumors of the nose include basal cell carcinoma, squamous cell carcinoma, and melanoma [9].

More specifically, there is also wide differential for lesions of the nasal septum. This includes congenital lesions (encephalocele, glioma, teratoma, dermoid, and epidermoid), granulomatous diseases (Wegener's granulomatosis and sarcoidosis), and neoplasms (basal cell carcinoma, squamous cell carcinoma, melanoma, adenoid cystic carcinoma, mucoepidermoid carcinoma, sarcoma, esthesioneuroblastoma, schwannoma, angiofibroma, and hemangioma) as well as infectious conditions (tuberculosis, syphilis, rhinoscleroma, leprosy, actinomycosis, aspergillosis, and mucormycosis) [15].

The origins of KA have not been completely elucidated but there are various causative theories which exist [2]. Its association with sun-exposed areas of the skin and its increased prevalence in patients with xeroderma pigmentosum have supported theories that exposure to UV light plays a role in the development of KA [2, 16]. Additionally, HPV-related DNA has been detected in KA specimens suggesting that there may be a viral etiology [17] while the increased presence of KA on or near skin graft sites supports a role for trauma as a causative factor [16, 18]. Even more, the increased prevalence of KA in patients following cyclosporine treatment suggest that immunosuppression may also play a role [19]. In the case of our patient, the presence of KA on the nasal septum can potentially be explained by the recent use of ranibizumab.

Ranibizumab is a vascular endothelial growth factor (VEGF) inhibitor that has been used for the management of macular degeneration since 2007 [20]. The cutaneous effects of ranibizumab are just starting to be elucidated, which include poor wound healing and mucocutaneous hemorrhage [21]. There has also been report of lichenoid keratosis with ranibizumab use [22]. Although ranibizumab has not been specifically linked to precancerous and cancerous skin lesions, other antiangiogenesis agents have been [20, 23]. Sorafenib, a multikinase inhibitor (including VEGF receptors), has been associated with actinic keratosis, focal squamous atypia, keratoacanthoma, and squamous cell carcinoma [23–25]. The mechanism is thought to be inhibition of the RAF kinase signaling pathway which results in keratinocyte differentiation and proliferation. Interestingly, these lesions have mostly been seen in men and have occurred in non-sun-exposed regions. Typically, skin lesions will arise between 2 weeks and 3 years after starting sorafenib [23]. The patient presented in the case report indicated that he began taking ranibizumab approximately several weeks to months prior to him noticing the lesion. Although it is difficult to prove associations in this isolated case, the role of ranibizumab causing cutaneous lesions should be further investigated.
